# Advances in Emerging Solar Cells

**DOI:** 10.3390/nano10030534

**Published:** 2020-03-17

**Authors:** Munkhbayar Batmunkh

**Affiliations:** Centre for Clean Environment and Energy, Griffith University, Gold Coast, Queensland 4222, Australia; m.batmunkh@griffith.edu.au

There has been a continuous increase in the world’s electricity generation and consumption over the years. Today’s energy requirements are principally met by burning fossil fuels. However, in addition to increasing fuel prices, greenhouse gas emissions caused by the fuel-burning process have become a serious issue. As such, the development of renewable and sustainable energy technologies is of great importance. Direct conversion of the sunlight into electricity using photovoltaic (PV) devices is now considered as a mainstream renewable energy source. According to the international energy agency (IEA) [1], the world’s total renewable-based power capacity is expected to grow by 50% between 2019 and 2024. Interestingly, solar PV accounts for more than 50% of this rise.

The PV market is currently dominated by technologies based on crystalline (poly + single) silicon. These silicon-based solar cells are a mature technology and can deliver a power conversion efficiency (PCE) of approximately 20% under full-sun illumination. Although significant reductions in the price of silicon PV cells have been observed, these technologies still suffer from high installation costs. Many scientists and researchers in the field of PV have paid particular attention to the development of a viable alternative PV technology. In this regard, emerging solar cells have received intense attention because these classes of solar cells, in comparison to traditional silicon PVs, promise to be less expensive, lighter, more flexible, and portable. Despite these great features, there are several challenges that restrict the possible commercialization of these technologies. This has led to significant efforts being focused on addressing issues associated with emerging solar cells. This Special Issue presents twelve excellent articles, ten research and two review papers, covering perovskite solar cells (PSCs) [2,3,4,5,6,7,8], heterojunction solar cells (HJSCs) [9], organic solar cells (OSCs) [10], dye-sensitized solar cells (DSSCs) [11], and PV materials [12,13].

The first report on organic–inorganic hybrid perovskite for solar cells was published in 2009 by Kojima et al. [14], and achieved a PCE of 3.8%. Since then, excellent achievements have been made in the PSC field and the certified efficiency of PSCs has now exceeded 25%, making them the fastest advancing PV technology. In this Special Issue, McDonald et al. [8] provided an excellent overview of PSCs and outlined the recent advances that have been made in nanoscale perovskites such as low-dimensional perovskites, perovskite quantum dots, and perovskite-nanocrystal based solar cells. Chang et al. [7] discussed the hot-carrier characteristics of perovskite light absorbers, which play a critical role in high efficiency PSCs. They also pointed out the practical issues hindering the development of highly efficient perovskite-based hot-carrier solar cells. The authors presented their own perspective on the future development of hot-carrier PSCs.

Although PSCs are very attractive and highly efficient, they suffer from several serious limitations. A typical PSC is fabricated using a transparent conductive electrode such as indium–tin oxide (ITO) and fluorine-doped tin oxide (FTO). However, these transparent electrodes are expensive and have natural brittleness and poor mechanical robustness. Two research articles in this Special Issue reported alternative transparent electrodes to the conventional ITO/FTO. Lu and colleagues [3] demonstrated that the composite electrode of silver nanowires and large area graphene oxide (Ag NWs/LGO) can exhibit comparable device performance to the standard ITO based PSCs. Chen et al. [5] designed a hexagonal Ni (30 nm)/Au (10 nm) mesh that showed a transmittance close to 80% in the visible light region and a sheet resistance lower than 16.9 Ω/sq. This metal mesh, when used in device fabrication, displayed a PCE of 13.88%, which was comparable to that of the ITO-based PSC.

Phenyl-C61-butyric acid methyl ester (PCBM) is the mostly commonly used electron transporting material in the p-i-n type (inverted) PSCs. However, the energy barrier at the interface between the PCBM layer and metal electrode limits the photogenerated charge extraction and thus results in reduced device efficiencies. In order to tackle this issue, Dong et al. [2] used a room temperature, solution processed Al-doped ZnO (AZO) as an interlayer between the PCBM and Ag electrode. The PSC device fabricated with an AZO interlayer not only exhibited a promising PV efficiency, but also showed excellent device stability. Incorporating additives into the perovskite has been proven to be a promising strategy to enhance the efficiency of PSCs. Wu et al. [4] explored the influence of adding water and potassium halides (KCl, KBr, and KI) into the PbI_2_ precursor solutions on the PV performance of PSCs. By co-doping with KI and water, they significantly improved the efficiency of CH_3_NH_3_PbI_3_ perovskite based solar cells. In PSCs, hole transporting materials (HTMs) play a critical role in selecting holes and transporting them to the conductive electrodes. High efficiency PSCs rely on expensive HTMs such as 2,2′,7,7′-Tetrakis[*N*,*N*-di(4-methoxyphenyl)amino]-9,9′-spirobifluorene (Spiro-OMeTAD) and poly[bis(4-phenyl)(2,4,6-trimethylphenyl)amine] (PTAA). In addition to their high costs, the devices fabricated using these HMTs suffer from poor stability in ambient conditions. Therefore, developing a novel HMT is of great interest. Wang et al. [6] designed a new type of HTM, named 4,4′-(9-methyl-9H-carbazole-3,6-diyl)bis(*N*,*N*-bis(4-methoxyphenyl)aniline) (CZTPA), as an alternative to the traditional Spiro-OMeTAD. This new HTM based PSC achieved a PCE of 11.79%, which was comparable to that (11.74%) of the non-doped Spiro-OMeTAD, while showing better stability in ambient conditions.

Solution-processed CdTe based HJSCs have attracted a great deal of attention from the PV community. However, the efficiencies of this class of HJSCs are still very limited. Mei et al. [9] developed an efficient approach to enhance the efficiency of CdTe/TiO_2_ HJSCs by inserting a thin layer of CdS nanocrystal between the CdTe and TiO_2_ layers. OSCs have many attractive properties such as high flexibility, solution processability, light weight, and simple manufacturing. In a typical OSC, poly(3,4-ethylenedioxythiophene):poly(styrenesulfonate) (PEDOT:PSS) is used as a HTM. However, the major drawback in using PEDOT:PSS in OSCs is the surface energy mismatch between the PEDOT:PSS and the active layer. To overcome this issue, Ramasamy et al. [10] used oleylamine-functionalized MoS_2_ in the PEDOT:PSS layer. By using this strategy, they observed a 15.08% enhancement in the device performance. DSSCs are an attractive emerging PV due to their eco–friendliness, ease of fabrication, and cost effectiveness. Designing a new type of dye molecule as a light harvesting material is still a hot area of research. Ambroz et al. [11] developed two bodipy dyes with different carboxylic acids on the meso-position of the bodipy core and used them to sensitize TiO_2_ photoelectrodes for DSSCs.

Exploring new synthesis methods, properties, and functionalization of PV materials is of great importance. Yang et al. [12] studied the electrical properties of 4H-silicon carbide (SiC) Schottky barrier diodes (SBDs) under high-dose electron irradiation. They used in-situ noise diagnostic analysis to demonstrate the correlation of irradiation-induced defects and microscopic electronic properties. Semiconductor SiC is widely used in electronic devices such as inverters, which deliver energy from PV arrays to the electric grids and other applications. Furthermore, Naffeti et al. [13] used a facile, reliable, and cost-effective metal assisted chemical etching method to fabricate highly crystalline vertically aligned silicon nanowires (SiNWs). SiNWs are widely used not only in solar cells, but also in other applications including lithium-ion batteries, sensors, electronics, and catalysis. SiNWs fabricated in this work [13] showed a strong decrease in the reflectance, demonstrating that these SiNWs are an excellent candidate for PV cells.

Finally, I believe that these articles will be of wide interest for the broad readership of the journal (*Nanomaterials*).
